# Frontal delta and theta power reflect strategy changes during human spatial memory retrieval in a virtual water maze task: an exploratory analysis

**DOI:** 10.3389/fcogn.2024.1393202

**Published:** 2024-05-01

**Authors:** Conor Thornberry, Sean Commins

**Affiliations:** Department of Psychology, Maynooth University, Maynooth, Ireland

**Keywords:** spatial navigation, spatial memory, virtual water maze, EEG, delta, theta

## Abstract

Brain oscillations in humans play a role in a wide range of cognitive processes, including navigation and memory. The oscillatory dynamics contributing to successful spatial memory recall in humans are not well-understood. To investigate specific oscillatory frequency bands during the recall process in human navigation, we recorded electroencephalographic (EEG) activity during a recall trial in healthy young adults (*n* = 15) following the learning of a goal location in a Virtual Water Maze task. We compared this to the activity during the same trial length, in a group of participants who did not learn a target location and navigated freely but were time-matched to the learning group (non-learning, *n* = 15). We compared relative power in Delta (2–4 Hz), Theta (5–7 Hz), Alpha (8–12 Hz), Beta (15–29 Hz), and Gamma (30–40 Hz) bands across the scalp. We found that delta and theta activity were greater during recall in our learning group, as opposed to our non-learning group. We also demonstrated clear suppression in the alpha band at posterior sites during memory-guided navigation compared to our non-learning group. Additionally, when goal-directed navigation switches to focused searching behavior, power becomes greater at the frontal region; with increases in the delta and theta bands reflecting this strategy change. There was also greater beta and gamma activity at posterior sites in our learning group. We discuss the results further in terms of the possible roles and functions of these oscillations during human navigation and hope this exploratory analysis can provide hypotheses for future spatial navigation and memory work.

## Introduction

Spatial memory is an important yet complex type of memory that allows us to encode and store information about our environment. A large body of work has indicated the importance of slow-wave oscillations such as theta (4–8 Hz) to spatial encoding and retrieval in both rodents and humans. These cellular (Eichenbaum et al., 1999; Moser et al., 2008; Colgin, 2020; Ormond and O'Keefe, 2022) and oscillatory (O'Keefe and Recce, 1993; Burgess and Gruzelier, 1997; Buzsáki, 2002, 2005; Burgess and O'Keefe, 2011; Buzsáki and Moser, 2013) findings typically derive from intracranial recordings. The cortical neurophysiology of spatial recall, including varying recall-based behaviors (such as directed and exploratory navigation) in humans, has not been readily examined in the literature.

Furthermore, evidence relating to the function of these oscillations and the human hippocampus suggests that low frequency oscillations from 1 to 12 Hz may be important for spatial navigation itself (Watrous et al., 2013). However, this is a very wide spectral range which has been linked to multiple other cognitive processes including episodic retrieval (Herweg et al., 2020; Vivekananda et al., 2021) and successful spatial working memory (Alekseichuk et al., 2016). Despite this, there is good evidence that oscillations in the 1–12 Hz frequency range are involved in both navigation and the recall of spatial locations. For example, Bohbot et al. (2017) found hippocampal oscillations between 4 and 12 Hz during both searching and recall during real-world navigation, as well as 1–8 Hz oscillations during virtual navigation. Similarly, it has been shown that low-frequency delta-theta oscillations in the hippocampus are responsible for encoding and retrieval of distance information (Vass et al., 2016). At scalp level, analysis of cortical activity by Jaiswal et al. (2010) showed theta oscillations lateralized to the right hemisphere during a virtual navigation task. Other researchers report oscillations around ~8 Hz being most prominent at the frontal and central midlines during spatial encoding and retrieval (Liang et al., 2018; Du et al., 2023).

Moreover, Du et al. (2023) recently found that frontal midline theta (4–8 Hz) increases accompanied by posterior (occipital and parietal midline) alpha (8–12 Hz) suppression are involved in encoding early in learning and are related to memory performance during virtual navigation. Similarly, Chrastil et al. (2022) revealed increased alpha during “active” virtual navigation but suppressed alpha at posterior sites with “guided” navigation. Alpha suppression may then reflect “passive” navigation without active recall or decision-making (as suggested by Du et al., 2023). Attentional demands during memory recall have also been commonly characterized by the suppression of alpha-band at posterior parts of the scalp (Klimesch, 1999; Foxe and Snyder, 2011). Finally, Chrastil et al. (2022) reported that beta power (12–20 Hz) was greater for correctly recalled decision-making and located at right frontal and left parietal channels. Furthermore, increases in beta (15–29 Hz) and gamma (>30 Hz) power have been reported in those with high levels of unsuccessful recall in a memory task, located near the medial temporal lobe and parietal areas (Waldhauser et al., 2012, 2015; Hanslmayr et al., 2016). It seems that increased high frequency oscillations are an indication of successful spatial memory formation.

Based on the above, we will perform an exploratory analysis of the oscillatory activity underlying immediate spatial memory during a recall trial in a virtual water maze task. We will examine scalp-EEG activity in participants who have successfully learned the task and are then required to recall the location of the goal. We will also compare this to the activity of participants who did not learn a goal location. We anticipate greater delta-theta activity and less alpha activity in our learning group compared to our non-learning group, with some differences between the groups in high frequency beta and gamma activity.

## Methods

### Participants

A total of 30 young adults (19 females, 11 males) aged between 18 and 45 (*M* = 23.03, SEM = ±1.014) who completed a learning phase from Thornberry et al. (2023) were included in this study. See Thornberry et al. (2023) for further information on sample size calculations. Only 30 of the original 50 participants completed a recall trial due to pandemic-related testing time restrictions (1.5-h maximum contact time). We lost eight due to time limitations, one due to not being comfortable with all the protective equipment, and 11 due to recording errors (e.g., electrode malfunction) that could not be fixed within the precautionary time limit. The power estimated for the comparison between the non-learning and learning groups with a Cohen's *d* of 0.8 and a sample size of 15/group at an alpha level of 0.05, was at least 0.69. All participants were right-handed. Participants were recruited via Maynooth University Department of Psychology and received course credit for their participation. The recall phase of this larger project and the use of human subjects with EEG was approved by the Maynooth University ethics committee (BSRESC-2021-2453422).

### Spatial navigation task

**Figure 1:**
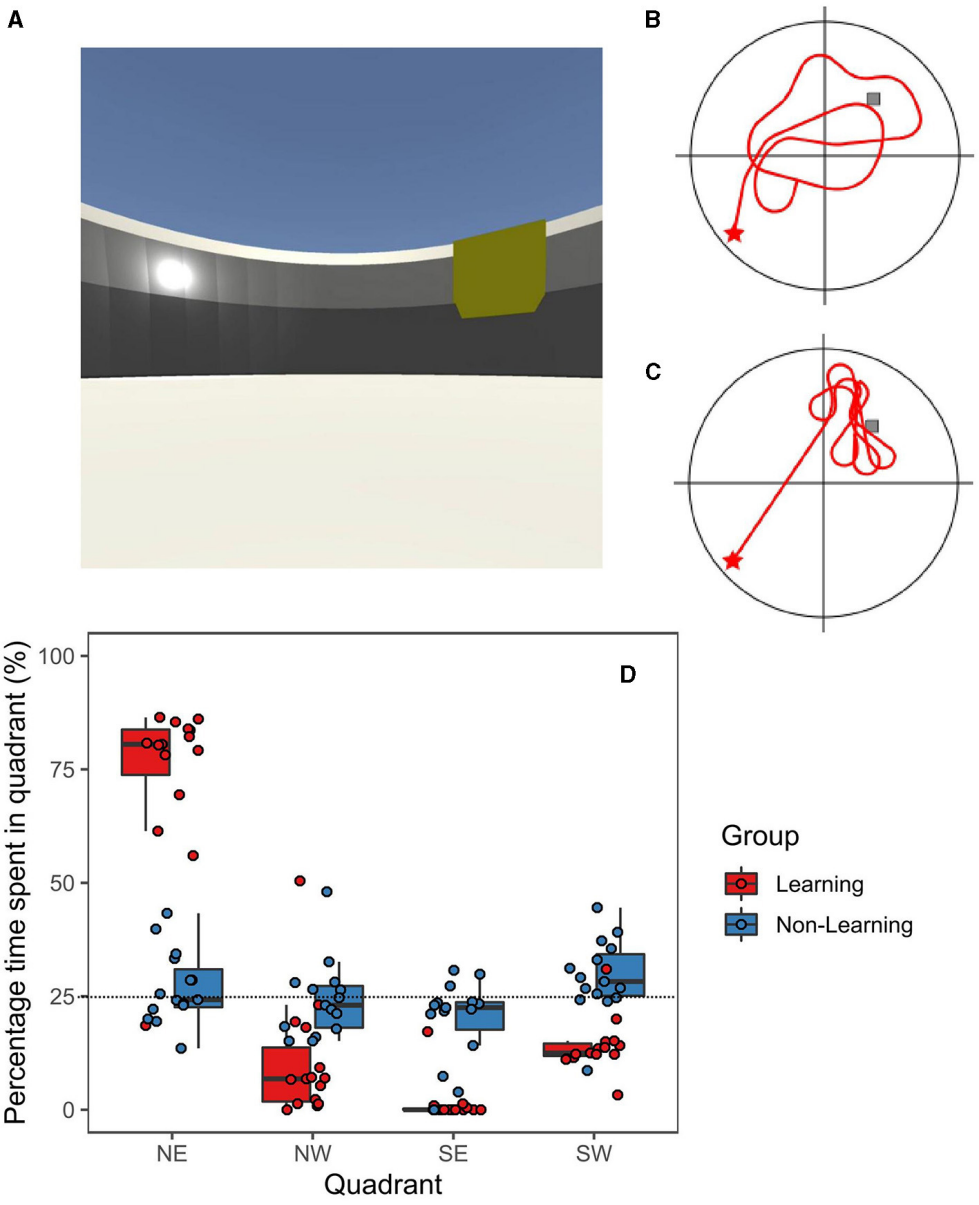
**(A)** Screenshot of the virtual maze with representative paths from non-learning group **(B)** and learning group **(C)**. Red star is starting position and gray box represents target. **(D)** Boxplot with individual data points displaying mean search percentage times for each quadrant (NE is target for learning group); Black horizontal bar represents the mean.

After the electrophysiological preparation, participants were seated 50 cm from the LCD computer screen on their own in a darkened, electrically shielded and sound-attenuated testing cubicle (150 × 180 cm) with access to a joystick for navigating. The spatial navigation task used was NavWell (Commins et al., 2020). The virtual maze setup consisted of a medium circular environment (taking 15.75 s to traverse) with two landmarks attached to the wall—a light and a large green square located in the NW (northwest) and NE (northeast) parts of the arena, respectively (see Figure 1A). Participants in the learning group (*n* = 15) were given 12 trials (60 s/trial) to learn the position of an invisible blue square in the middle of the northeast quadrant (the goal). It became visible when walked over and was 15% of the total arena size. Participants in the non-learning group (*n* = 15) were also given 12 trials. For this group there was no goal present; however, the environmental setup was exactly the same as the learning group. In addition, the time spent moving on each trial matched the average time for the corresponding trial for the learning group. All participants started from N, S, E and W positions in a pseudo-random order. Once the learning phase was completed all participants took a 10–15-min break. Following this, participants were given a single 60-s recall trial. Participants in the learning group were required to recall the location of the target. However, for this trial, the target was removed. Those in the non-learning group were similarly allowed 60 s to move around the arena. All participants started from the same novel south-west (SW) location for this trial. The % time (of the 60 s) spent in the goal and the other three quadrants of the arena was used to measure recall.

To breakdown and analyse different elements of behavior during the recall phase, we exported the *x*-*y* co-ordinates across time for each participant from NavWell. The *x*-*y* co-ordinates are recorded and stored in a *JSON* file every 0.25 s as a participant traverses the arena. Therefore, we first plotted the *x*-*y* co-ordinates in a plane that displayed the entire path trajectory. Based on these trajectories we derived a clear discrimination of behavior, based on each quadrant and participants starting position. We plotted the *x*-*y* co-ordinates for each individual participant onto the same graph.

### EEG recording, preprocessing and analysis

EEG data was acquired using a BioSemi ActiveTwo system (BioSemi B.V., Amsterdam, Netherlands) providing 32 Ag/AgCl electrodes positioned according to the 10/20. Electrode impedance was checked and adjusted to be below < 20 Ω before recording began again. Analog event signals were sent only once when participants began their trial. Four electrodes (EXG1–EXG4) were positioned on the face to capture EOG artifacts. Raw EEG data were again sampled at 1,024 Hz but down-sampled offline to 512 Hz. Continuously recorded EEG data were analyzed offline in MATLAB R2021B using scripts in combination with the Brainstorm package (Tadel et al., 2011). A 1 Hz high-pass filter and a 40 Hz low-pass filter were applied. Independent Component Analysis (ICA) was performed to remove and correct artifacts, namely eye movements, blinks, and muscle artifacts. For this analysis, the entire continuous recording was then epoched into 2-s epochs, producing 30 epochs per participant. These data were visually inspected for bad segments and bad electrodes, which were then removed. Bad electrodes were interpolated if possible (*n* = 2). Epochs with voltage steps above 100 μV or peak-to-peak signal deflections exceeding 200 μV within 2-s intervals were automatically rejected. We had a rejection rate of ~8% of the total epochs produced. EEG data were then re-referenced to the average of the 32 electrodes.

As this was an exploratory analysis, we investigated five frequency bands: Delta (2–4 Hz), Theta (5–7 Hz), Alpha (8–12 Hz), Beta (15–29 Hz), and Gamma (30–40 Hz). Power spectra were computed on artifact-free epochs for each participant. We used Hanning windows of 2-s with a 50% overlap using Welch's method for all electrodes. This resulted in a spectrum with frequency resolution of 0.5 Hz. Power was computed using the underlying short Fast Fourier Transform (sFFT) with a linear frequency distribution of 1:1:40. Relative power within these bands was then computed to reduce inter-subject variability in the power calculations. For the behavior-based EEG analysis, we split our EEG recording into relevant timestamps to examine EEG activity during these different navigational behaviors. We then divided artifact free 2-s epochs up into behavioral “phases” based on time from event trigger for trial start. All epochs in each group and phase, were averaged together following computations to compute one Power Spectral Density (PSD) per individual which were then compared at the group level.

### Statistical analysis

Statistical analyses and visualization of the behavioral data were performed using a combination of JASP (version 0.15) and *R* software version 4.0.2 (R Core Team, 2013) with the tidyverse and ggplot2 package. Statistical exploration of the EEG data across the scalp was performed using Brainstorm in MATLAB 2021b, comprising of two-tailed non-parametric independent *t*-tests with 5,000 permutations and a *p*-threshold of 0.05. We assumed equal variance and corrected for multiple comparisons in EEG data using an FDR (False Discovery Rate) correction (with an average threshold of 0.01). All data were combined for behavior-matched EEG analysis. For descriptive statistics, we reported Mean Difference (MD), Mean (M) and Standard Error of the Mean (SEM).

## Results

### Behavioral results

Firstly, we compared the ages of the learning group (*n* = 15, 10 females) to the non-learning group (*n* = 15, nine females) to confirm our groups were well-matched (*M* = 23.60, SEM ± 1.66 and *M* = 22.47, SEM ± 1.21). We report no significant difference between the groups [*t*_(28)_ = 0.55, *p* = 0.59, Cohen's *d* = 0.202]. Participants in the learning group improved across the 12 trials [*F*_(4.5, 58.5)_ = 7.54, *p* < 0.001, η^2^ = 0.32], as participants in the non-learning group were matched for time this was not analyzed. Further details on the learning phase and its neural correlates can be found in Thornberry et al. (2023). For the recall trial, the percentage time spent searching in each quadrant of the arena (including the target quadrant, i.e., NE) was recorded for both groups. The data were analyzed using a 2 (Group) X 2 (Gender) X 4 (Quadrant) repeated measures ANOVA. We reported a significant main effect of Quadrant [*F*_(1.2, 30.2)_ = 12.21, *p* < 0.001, η^2^ = 0.19]. We reported no significant between subjects effects for either Group [*F*_(1, 26)_ = 1.50, *p* = 0.23] nor Gender [*F*_(1, 26)_ = 1.50, *p* = 0.23]. However, we revealed a significant interaction effect for Quadrant X Group [*F*_(1.2, 30.2)_ = 13.69, *p* < 0.001, η^2^ = 0.22] but found no significant interaction effect for Quadrant X Gender [*F*_(1.2, 30.2)_ = 1.24, *p* = 0.28] nor a significant three-way interaction effect (*p* = 0.26).

Focusing on the goal quadrant, we ran Tukey-corrected *t*-tests to investigate the reported interaction effects. The learning group's percentage of time spent in NE (M = 78.12%, SEM ± 2.30%) was significantly greater (MD = 51.72%, *t* = 4.84, Cohen's *d* = 1.84, *p* < 0.001) compared to the time spent searching there by the non-learning group (M = 26.9%, SEM ± 2.1%). Additionally, the learning group spent significantly more time searching in the goal quadrant (NE) than all other quadrants; NW (M = 7.97%, SEM ± 1.8%, *p* < 0.001), SW (M = 12.59%, SEM ± 0.89%, *p* < 0.001) and SE (M = 1.32%, SEM ± 1.14%, *p* < 0.001). Importantly, time spent searching in the goal quadrant (NE) in the non-learning group, did not differ from any of the other quadrants: NW (M = 24.22%, SEM ± 2.18%, *p* < 0.99), SW (M = 29.2%, SEM ± 2.16%, *p* < 0.999), nor SE (M = 19.64%, SEM ± 2.37%, *p* < 0.758). Therefore, all quadrants for the non-learning group were near chance levels (25%) and did not differ from each other. Directed searching was displayed in the learning group (Figure 1C), who spent a statistically significant amount of time searching in the goal quadrant compared to the non-learning group (see Figures 1B, D).

### EEG results

**Figure 2:**
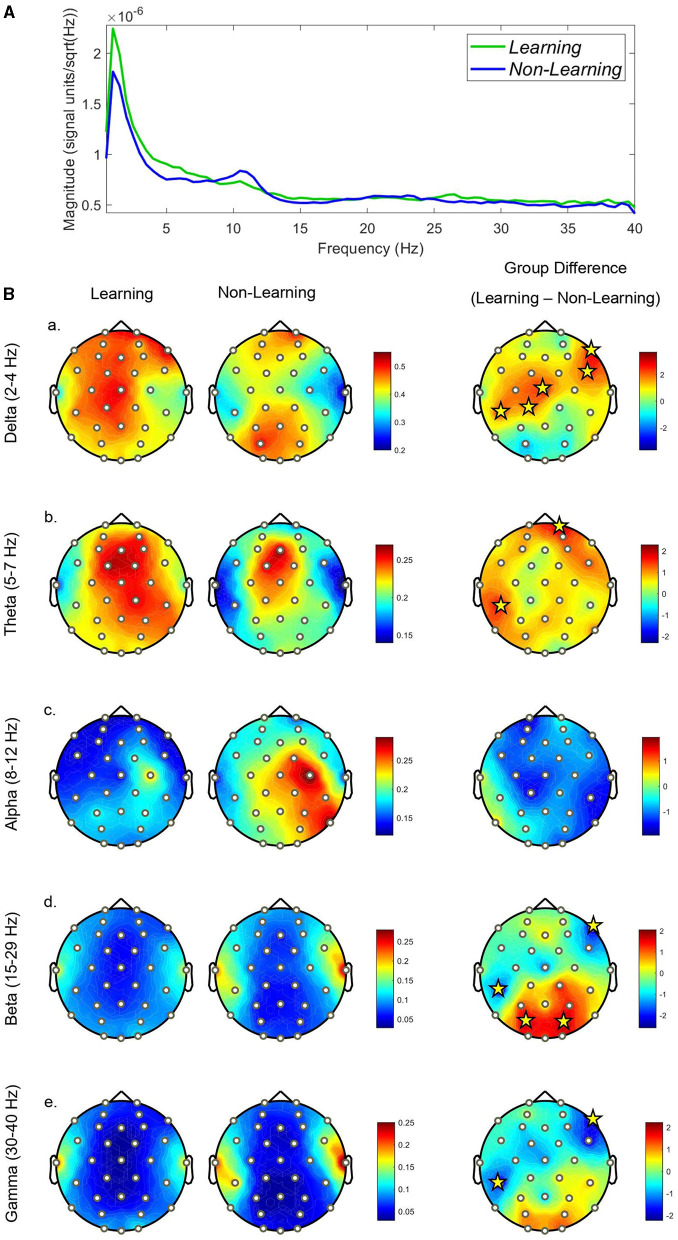
**(A)** PSD graph displaying mean scalp power for each group across the full recall trial. **(B)** Topographical plots displaying each groups relative power distribution across the scalp at each frequency-band (a–e). Significant electrodes are displayed with yellow stars. The scale in relative power (%), whilst the scale for the differences is displayed in *t*-values.

We ran FDR-corrected permutation *t*-tests to reveal significant group level differences at all electrode sites and frequency bands (at an alpha level of 0.05 with 5,000 permutations). We first analyzed the entire recall trial, which results in the comparison of a total 438 epochs for the learning group and 450 epochs for the non-learning group—which were then computed as a PSD at an individual level and compared. Results are reported as topographies in Figure 2B. We reported that topographical distribution of power was significantly greater in the learning group in the Delta band (2–4 Hz) at frontal and central sites including CP5, Cz, F8, FC6 (*t* = 2.89, 2.94, 3.76, 2.85; all *p* < 0.01, respectively) and CP1 (*t* = 2.21; *p* < 0.05). Similar significant increases in Theta (5–7 Hz) are found at frontal and central sites for the learning group, with CP5 and FP2 as significant (*t* = 2.06, 2.42; both *p* < 0.05 respectively). Within the alpha range (8–12 Hz) we report no significant differences at any electrode site, but a large widespread activation over central and parietal sites are observed for the non-learning group, with most of the activation near the right motor areas (site C4). Whilst a small activation is also seen in the learning group, it is not as prominent as the non-learning group, suggesting a suppression. In Beta (15–29 Hz) we reported significantly greater power in the learning group compared to the non-learning group at occipital sites, with significance at sites PO3 and PO4 (*t* = 2.28, 2.24; both *p* < 0.05, respectively), whilst reporting significantly isolated, but lower power at sites CP5 and F8 (*t* = −2.54, −2.488; both *p* < 0.05, respectively). Finally, for gamma (30–40 Hz) we report similar findings to beta, but not as marked, losing significance at sites PO3 and PO4, but retaining significance for CP5 and F8 (*t* = −2.22, −2.26; both *p* < 0.05, respectively). We show some distinctive differences across all bands, which are reflected in the PSD plot in Figure 2A.

### Behavior-matched EEG dynamics

**Figure 3:**
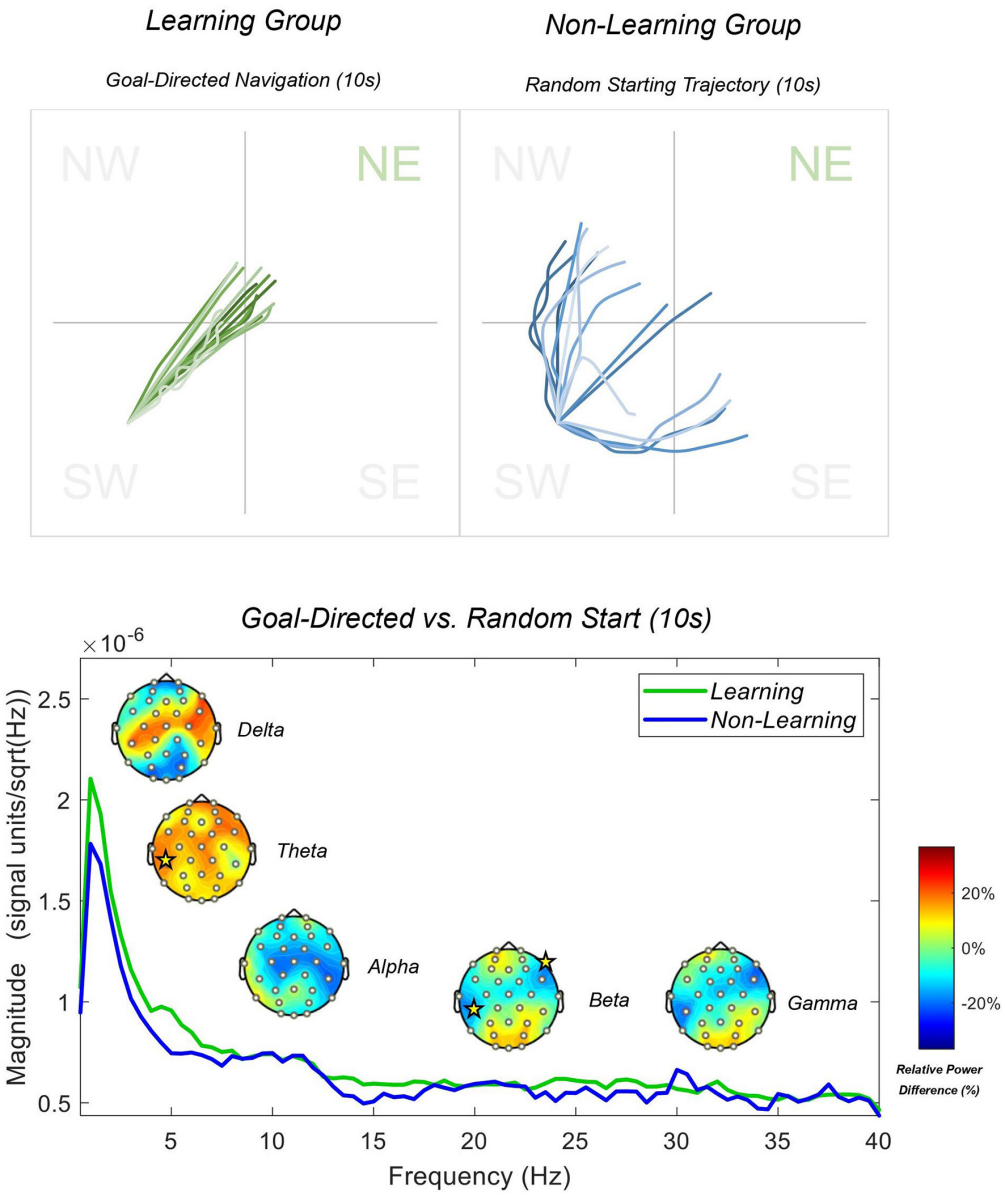
Learning and non-learning group paths generated via *x*-*y* co-ordinates from the first 10 s of the recall trial are displayed above. PSD spectrum during the first 10 s of the recall trial is displayed below. Topographies display the results of between-subjects permutation *t*-tests. Yellow stars indicate electrode sites with a significance level of 0.05 or less. Topographies are displayed in relative power differences, and the PSD is plotted in magnitude with a resolution of 0.5 Hz.

In the classic Morris water maze (MWM) paradigm, during the probe trial of animals who have successfully learned the task, there is an initial goal-directed searching behavior followed by focused but wider searching behavior (Harvey et al., 2009; Rogers et al., 2017; Nyberg et al., 2022). We expected to see evidence of these two searching behaviors during our probe trial in our learning group: goal-directed navigation, followed by focused-searching. Furthermore, we would expect to see purely random searching behavior in our non-learning group throughout the trial. However, to capture the true dynamics of the search path across time, we exported the *x*-*y* coordinates from the NavWell system for each participant (see Figure 3 below). Following this, based on the analysis of screen-recorded probe trials from participants, and the short escape latencies from the learning phase within the learning group, we split our trajectories into the first 10-s (which captured most of the goal-directed navigation) and the final 50-s (which captured most of the focused searching behavior in the NE quadrant) for the learning group. For the non-learning group, the first 10-s showed that participants headed off in random directions and then continued to move around the entire arena for the rest of the trial in a random fashion.

### Goal-directed vs. random behavior

**Figure 4:**
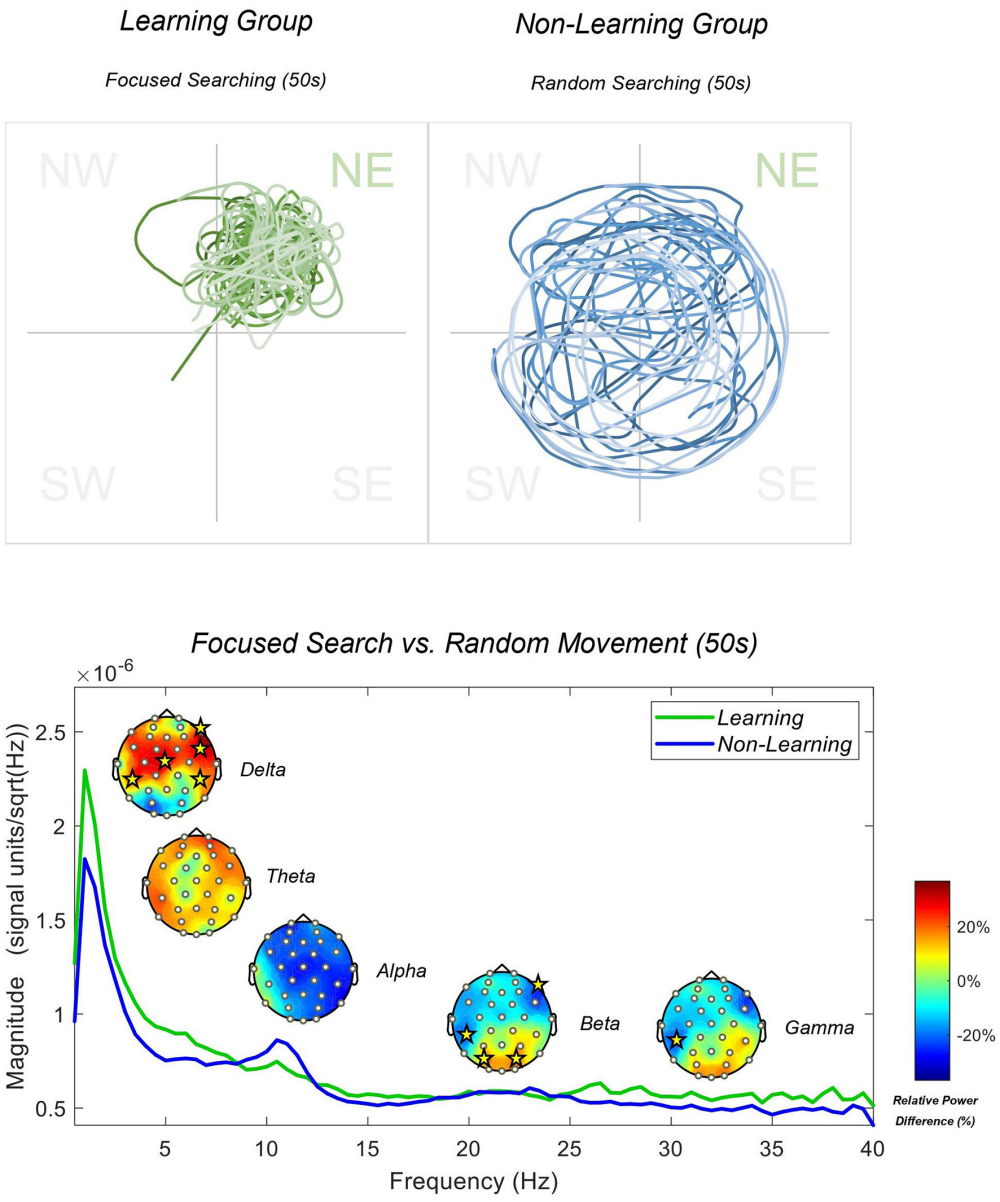
Learning and non-learning group paths generated via *x*-*y* co-ordinates from the last 50 s of the recall trial are displayed above. PSD spectrum during the first 10 s of the recall trial is displayed below. Topographies display the results of between-subjects permutation *t*-tests. Yellow stars indicate electrode sites with a significance level of 0.05 or less. Topographies are displayed in relative power differences, and the PSD is plotted in magnitude with a resolution of 0.5 Hz.

To examine difference between groups during each behavior, we utilized a standard Welch's PSD with an underlying sFFT linear function of 1:1:40. Individual PSD's were produced from 75 epochs per group for the initial 10 s phase, and then 363 epochs for the learning group and 375 for the non-learning group for the final 50 s phase. We present full-scalp power differences between the groups in Figure 3 (initial 10 s) and Figure 4 (final 50 s) below. For this phase, we compared relative frequency power differences between groups using an independent permutation *t*-test with 5,000 permutations as implemented previously. Again, we utilized relative power as we are interested in the distribution of power within the frequency bands between the groups. Additionally, this calculation generated better between-group comparable data that is standardized and accounted for slow-drifts, artifacts and noise that may influence between-group analysis. The two groups may also have differing overall levels of absolute power (e.g., absolute alpha power changes with age) and therefore relative power provides a correction for this when comparing across groups (see Jabès et al., 2021, for further information). Figure 3 shows that participants headed directly toward the goal (learning group), with increased delta and theta power, with significant increases in theta at the CP5 electrode (*t* = 2.117 and *p* < 0.05), when compared to participants that headed in a random direction (non-learning group). We reported less alpha power across central sites, but none reaching significance. We further reported greater beta and gamma power at anterior and posterior sites, but significantly less power at sites CP5 and F8 for beta only (*t* = −2.19, −2.11; both *p* < 0.05, respectively). Figure 4 shows that participants that had searched with a focus on the NE quadrant showed even greater increased delta and theta power, with significant central electrodes CP5 and Cz (*t* = 3.16, 3.02; both *p* < 0.01) and frontal electrodes FC6 (*t* = 2.87; *p* < 0.01) and F8 (*t* = 3.90; *p* < 0.001) in delta compared to the non-learning group. In addition, as reported above, this group showed higher frequency activity in the beta and gamma bands within the parietal and occipital regions (significant at PO3 and PO4 for beta only; *t* = 2.39, 2.34; both *p* < 0.05, respectively), compared to the non-learning group that moved randomly throughout the entire arena. We also reported significantly less beta in our learning group at sites CP5 and F8 again (*t* = −2.56, −2.50; both *p* < 0.05, respectively), with CP5 only significant (*t* = −2.37; *p* < 0.05) in the gamma band. Finally, the learning group showed lesser alpha power (~11 Hz) during random movement, which was not observed in the non-learning group (see Figure 4).

## Discussion

The current study set out to perform an exploratory analysis of the oscillatory activity involved in recall of a goal location. Based on previous literature, we predicted a couple of findings. Firstly, we expected low-frequency oscillations (2–8 Hz) to play an important role in the recall process. Additionally, we expected alpha oscillations to be suppressed in the learning group, but not in the non-learning group. Furthermore, we expected to demonstrate some high frequency (>15 Hz) differences between the groups. These predictions were generally observed.

### Delta-theta oscillations

We reported greater delta (2–4 Hz) and theta (5–7 Hz) power in our learning group compared to our non-learning group during the recall trial. Greater power was topographically located at frontal and central sites but appears prevalent across the scalp. Our results support the suggestion that low-frequency oscillations in humans (2–8 Hz) are involved in successful memory-guided navigation (Greenberg et al., 2015; Alekseichuk et al., 2016; Crespo-García et al., 2016; Bohbot et al., 2017; Liang et al., 2021; Vivekananda et al., 2021). The widespread activation of delta and theta in our learning group is contrasted with only isolated delta and theta activity in our non-learning group. This idea is further supported by our behavior-based analysis on our low-frequency oscillations. During goal-directed navigation at the start of the trial, we reported greater theta power across the scalp, and greater delta power at central regions compared to our non-learning group's random starting trajectory. However, as participants switched to focused searching, we reported significantly greater frontal and central widespread delta power compared to the random searching of the non-learning group. Many of our significant electrodes were in the frontal right hemisphere of the scalp. Our results align with previous findings of the involvement of low-frequency oscillations within the range of 2–8 Hz being involved in successful spatial memory *during* navigation (Nishiyama et al., 2002; Buzsáki and Moser, 2013; Watrous et al., 2013; Greenberg et al., 2015; Alekseichuk et al., 2016; Bohbot et al., 2017; Delaux et al., 2021; Liang et al., 2021; Miyakoshi et al., 2021; Chrastil et al., 2022; Lin et al., 2022; Du et al., 2023). Furthermore, our evidence suggests that theta supports the overall initial goal-directed retrieval and navigation, whereas delta (or lower frequency oscillations) become involved in subsequent focused searching, or when greater cognitive demand is placed on spatial memory systems.

These findings and interpretation map onto previously discussed intracranial data from humans, that illustrate successful associative retrieval results in increased low frequency oscillations (< 5 Hz), whereas low-frequency oscillations between 5 and 9 Hz seem to be increased during encoding (Lega et al., 2012; Bohbot et al., 2017; Kota et al., 2020). It could be argued here that the initial phase of goal-directed navigation does not require the recollection of learned associations (i.e., cue and goal) but instead incorporates retrieval of a place, using theta oscillations engaged during learning as found in our previous work (Thornberry et al., 2023). When this retrieval strategy fails, low-frequency oscillations are further recruited to retrieve learned associations between the goal and other environmental stimuli (e.g., landmarks), to perform memory-dependent focused searching. Moreover, the involvement of the frontal midline during a virtual navigation task supports the involvement of this region in active spatial navigation, reported by multiple other studies (Mitchell et al., 2008; Liang et al., 2018, 2021; Chrastil et al., 2022; Lin et al., 2022; Du et al., 2023). Nonetheless, it is difficult for us to report a *specific* role of increased frontal delta and theta in memory retrieval. However, it is clearly involved to a greater extent when a task has been learned, and delta in particular is heightened when applying memory based as opposed to random spatial search strategies.

### Alpha oscillations

We reported markedly less alpha (8–12 Hz) power in our learning group compared to our non-learning group across the entire duration of the recall trial, however no electrode site reached significance following correction of multiple comparisons. The non-learning group show distinctly heighted alpha across the right hemisphere, with some concentration on central electrodes (C4 in particular, a sensorimotor electrode ipsilateral to the hand moving the joystick—all participants were right-handed). Some right-posterior electrodes also possess heightened alpha, with the learning group showing C4 activation, but not to the same extent. Furthermore, the non-learning group show increased alpha (centralized at ~11 Hz) during random searching, particularly as the trial progressed. This was not observed for the learning group.

The role of alpha in attention has been well-documented. Decreases typically reflect an idle or focused state. Increases in this rhythm have been linked to difficulty focusing due to external, irrelevant or competing stimuli (Foxe and Snyder, 2011). Our reported high-relative power in our non-learning group may indicate a lack of focus or attention during random exploration. As the trial progresses, this lack of focused attention increases. Alpha desynchronization is typically associated with focused and controlled information processing (Klimesch, 2012). Lower alpha power has been reported during spatial attention tasks (Thut et al., 2006; Capotosto et al., 2009; Li et al., 2021). However, even with distractions, alpha power has recently been reported to decrease (Fodor et al., 2020). Our results from the learning group suggest that alpha may be suppressed to a greater extent, and may index engaged memory-guided attentional mechanisms, facilitating focus and access to the memory of the goal. However, due to issues with sample size, correction for multiple comparisons and lack of significant electrode sites, these results should be interpreted with caution. Nevertheless, based solely on descriptive and observed differences, one could argue that using memory-guided attention to navigate, suggests that this group have less effortful and more fixated attention—as their search strategy is memory-guided and place focused. Therefore, this interpretation would support the concept proposed by Du et al. (2023), that increased alpha reflects competing spatial cues. The non-learning group had more conflicting information processing and/or a lack of focused attention. Furthermore, these match those of Chrastil et al. (2022) who reported elevated alpha power during undirected navigation with multiple decisions, as opposed to suppression of alpha power in a guided navigation group, who had no competing spatial information. Despite this, we report no significant electrode sites. It is entirely possible, that analysis of relative power within each frequency band, and the frequency definitions assigned to those bands, may have reduced the likelihood of reporting a significant difference. We would recommend future work calculates baseline activity or utilizes individual alpha frequency (IAF) for each participant (Bazanova, 2012; Bazanova and Vernon, 2014). It is possible that the expected differences in visual attention between the groups, have been picked up in higher frequency bands (as discussed below) due to differing individual levels of alpha and a lack of an individual controlled baseline. Furthermore, gradually increasing alpha in both groups is perhaps related to increased mind-wandering (Compton et al., 2019; Kam et al., 2022) or a reduction of focused-attention and motivation.

### Beta-gamma oscillations

We reported significantly elevated posterior beta (15–29 Hz) power in our learning group compared to our non-learning group across the entire trial, with significance at PO3 and PO4 electrodes, which are located around the occipital lobe. We reported similar results for gamma (30–40 Hz) during directed searching but to a less degree with no significant electrode sites being found. Overall significant decreases were found at right frontal and left parietal sites as were found in beta. Significantly stronger posterior beta in our learning group, was only found during the focused searching and not during the initial start trajectory. This may indicate that beta plays a role in more directed visual scanning, as opposed to direct route following. However, it may also represent differences in visual attention between the groups, which is typically detected at high-frequency alpha rhythms (Sauseng et al., 2005; Peylo et al., 2021). Several studies also report enhanced beta and gamma power during visual and spatial working memory retention and maintenance (Park et al., 2011; Roux et al., 2012; Proskovec et al., 2018) particularly located at posterior parts of the scalp (Medendorp et al., 2007; Honkanen et al., 2015). Beta power at parieto-occipital regions has previously been reported to increase with memory load (Tuladhar et al., 2007). Roberts et al. (2013) found using scalp EEG that beta and gamma activity increased during the maintenance of spatial, but not temporal working memory. Therefore, we suggest that greater beta power in our learning group at parieto-occipital sites may be related to memory maintenance or memory load, and possible memory-guided visual search, which is not used in the non-learning group.

### Limitations, future recommendations, and conclusions

The primary limitation is the low sample size used in this analysis. At the time, participants could not stay for longer than 1 h in the lab due to COVID-19 restrictions in place at the time. This meant that some participants could not perform their recall trial after learning the task. Therefore, the sample is smaller, and predominantly female. Future studies should try to balance for gender and increase participant numbers. Based on this and the exploratory nature of the study—we advise that any results be interpreted with caution, even though our original sample size was well-justified. We hope the current exploratory study is sufficient to generate new approaches to studying oscillations during active navigation. Furthermore, increasing electrode channel numbers (from a 32-channel cap) would allow accurate source analysis (Michel and Brunet, 2019). Though more ecologically valid, our task does lack vestibular and sensorimotor feedback during the 2D navigation. Considering we used the standard paradigm for the Morris water maze (Vorhees and Williams, 2014) and the virtual water maze (Thornberry et al., 2021) we could only give participants a probe/recall trial once. Giving any more trials would not actually examine memory. Furthermore, the nature of the virtual water maze (VWM) tasks means that memory-guided searching behavior is elicited continuously. As a result, once a participant was engaged on their chosen path, there was no time window in between segments to serve as individual baselines to which the data could be normalized. As the data is continuously collected for the 60 s trial, statistical and oscillatory data only allowed for interpretations about the relative changes observed between the two groups and behavioral conditions. This also meant our data had to go through a rigorous preprocessing procedure as signal-to-noise ratio could be dramatically influential. Future research should include a baseline data collection to facilitate changes from idle cognitive states to spatial memory “recall” states. Nonetheless, greater anterior low-frequency oscillations, suppressed posterior alpha and greater occipital beta oscillations are associated with successful spatial memory retrieval and memory-guided navigation behavior. Both learning and non-learning groups were engaged in active exploration, allowing this study to decouple memory-guided navigation from both sensorimotor integration and exploratory processes. This exploratory work is an essential step toward understanding the role and function of oscillation patterns in active virtual navigation.

## Data availability statement

The datasets presented in this study can be found in online repositories. The names of the repository/repositories and accession number(s) can be found below: https://osf.io/a5z9k/.

## Ethics statement

The studies involving humans were approved by Maynooth University Ethics Committee: The Biomedical and Life Sciences Research Ethics Subcommittee (BSRESC-2021-2453422). The studies were conducted in accordance with the local legislation and institutional requirements. The participants provided their written informed consent to participate in this study.

## Author contributions

CT: Conceptualization, Formal analysis, Investigation, Methodology, Software, Visualization, Writing – original draft, Writing – review & editing. SC: Conceptualization, Methodology, Resources, Supervision, Writing – review & editing.
